# Current Hospital Topics

**Published:** 1907-01-26

**Authors:** 


### CURRENT HOSPITAL TOPICS.

### Shortage of Resident Surgeons.

At the present time there is a distinct shortage in the number of medical applicants for house appointments. The applicants have not been so few since 1900, shortly after the outbreak of the South African War, which naturally drew off a great number of young practitioners. Several of the London general hospitals have had difficulty in finding suitable candidates for resident posts, and the provincial hospitals are said to have suffered even more severely. At the Lincoln County Hospital it has been the custom for many years, to pay the house surgeon £100 per annum. As, however, the weekly board do not find the class of candidates who offer themselves for election, very satisfactory, they have decided to raise the salary to £125 with the hope of attracting a better stamp of practitioner.

### Out-Patient Reform.

The Norfolk and Norwich Hospital has issued the following statement, embodying the new regulations which the Governors have made, with a view to prevent abuse as much as possible. We shall watch this scheme with considerable interest, and it is encouraging to find that, an important provincial hospital should have, spontaneously, taken action in the direction of hospital reform.

"The General Board of Governors having decided to make the out-patient department free, the out-patient recommendations are no longer issued. In future any person requiring medical attendance, and who is unable to pay for adequate treatment, can apply at the hospital on Saturdays at 10.30 A.M., and he or she will be seen. If in the opinion of the physician or surgeon, after examination, the case is one requiring special treatment, or if the patient is unable to procure the requisite attendance elsewhere, treatment will be provided in the out-patient department of the hospital. If, however, it is considered that the patient's malady does not demand special or hospital treatment, he or she will be so informed and referred for treatment elsewhere. Those applicants who have not made any provision for medical attendance in the case of sickness will be advised as to the best means of securing it for themselves. Governors and subscribers are invited to send to the hospital any needy person requiring medical attendance, and any information as to the circumstances of such person addressed to the admission committee will be much appreciated. It is thought that this change will be to the advantage of out-patients, both county and city."

### The Inspection of Hospitals.

Hospitals nowadays have an increasing attraction for the majority of residents in the districts where they may be situated. A new hospital building excites very general interest, and it is a wise step for the authorities to throw all new hospital buildings open to public inspection, before the patients are received. The new cancer hospital, known as the Caird Hospital, Dundee, is a case in point. During the holidays the buildings were thrown open to the public on two successive days. The attendance of visitors was very large, all classes being represented. Amongst those present at almost any hour on each day might be seen city merchants, medical men with their wives, ordinary citizens of all types, in addition to many working folk, and there was not one visitor who did not show the keenest interest and a desire to understand the nature of the different departments and the accommodation provided. Dr. Fraser and Miss Duff were indefatigable in their endeavours to gratify everybody, and they were well seconded by the sister in charge and a large body of nurses. If we may judge by the scene presented, we should think it is probable that no member of the staff, ever before had so many questions to answer, as on this momentous occasion. Apart altogether from the direct benefits which are likely to be conferred by the Caird Hospital, the interest which it has excited, locally, cannot fail to do a great amount of good in many ways.

### Business Methods in Hospital Management.

It is not sufficiently realised by many hospital managers, that, after all, the conduct of a hospital is a great business, which should be conducted on business principles, so that the maximum of economy and efficiency may be everywhere present, throughout the system. This point is fully recognised by the committee of the Royal Victoria Infirmary, Newcastle-on-Tyne. The opening of the new infirmary buildings, containing increased and improved accommodation, will entail an increased expenditure of £10,000 a year. A suggestion has been made by one of the Governors, and is being widely followed, that all the existing subscribers should be asked to double their subscriptions. Those of them who are so prosperous, as to make compliance with this suggestion a matter of relative unimportance, may be expected to respond heartily to this appeal. When, however, we realise that the population of Newcastle is upwards of a quarter of a million, and that only 890 people gave an annual subscription to the infirmary during 1906, it will be seen that, the committee will be well advised to make an earnest and continuous attempt to awaken the great mass of relatively prosperous people above the artisan class, who, for the most part, fail to contribute anything, regularly, to the relief of their poorer neighbours. We believe that, if the responsibility of making a contribution of this kind, in the days of health in the cause of the sick, could be brought home to the middle-class residents in Newcastle, the £10,000 required would be subscribed twice over within the year. It is greatly to the credit of the working-class population that their direct contributions should have increased from £3,592 in 1895 to £5,647 in 1905 and to an estimated £8,600 in 1906. The workmen are determined nevertheless to raise still more money. The miners in Durham and Northumberland, who have been presented with a business statement by the hospital authorities, showing that the contributions received from owners and workmen in both counties, are considerably below the maintenance cost of the miners who have been treated in the infirmary, have formed the resolution to wipe out these deficits, amounting to upwards of £2,000. They have further determined to give, in addition, a substantial contribution to the general work of the charity. We hope that the splendid example set by these self-respecting miners will stir up the middle-class residents, who, so far, have shown little disposition to bear their share in maintaining Newcastle's great palace of pain, which is one of the noblest institutions of the North.

### Patients' Payments in Infectious Hospitals.

IT is essential that, wherever there is a hospital for infectious diseases maintained by a local authority, that hospital shall be kept efficient for immediate use throughout the whole year. Further, it must be so organised as to have available an efficient staff, to deal with every case of infectious disease which may be sent in from time to time. It is one of the blots upon our local government system, throughout the country, that there are far too many so-called hospitals for infectious diseases which are not so organised, and are in consequence unfit places to send a patient to, when suffering from an acute fever. Only, recently, a well-known member of the profession publicly stated, that one of his patients, whom he sent in good faith to a hospital of this latter type, had suffered in health and had had his life imperilled by the disgraceful condition of the so-called infectious hospital. The practitioner was right in refusing to send any other case there, until the authorities provided an adequate establishment, and kept it always ready and efficient for the admission of patients. We wonder that the ratepayers, any one of whom may suffer grievous injury, and some of whom may even lose their lives, by the neglect of the authorities to provide an adequate infectious hospital, should show so much supineness by tolerating a state of affairs such as that referred to. The ratepayers should everywhere form a committee to inspect periodically, and to keep a very close eye upon, the condition and administration of the infectious diseases hospital, in their neighbourhood. It was urged, at the Halstead Union District Council, recently, that no charge should be made for the treatment of patients in the infectious hospital. The grounds for this contention were, that such a hospital benefits to a greater extent the whole of the residents by preventing the spread of infectious disease, than it benefits the patients, and so the former should bear the whole cost of treatment and upkeep. The law is, that, patients, who can afford to pay, should be made to pay for their treatment, and Mr. Wallace, the chairman of the Hospital Committee, was wise in insisting that payment should be enforced wherever possible. No delay in admission need be caused by such a regulation, for it is the duty of the health authorities to send in the patients, and the duty of the hospital authorities to ascertain the circumstances of the family, after admission, and to fix the rate of payment in accordance with the circumstances of each case. The experience of Halstead appears to be that the poorer residents are quite ready to pay, but that the middle-class mechanics, who are well able to do so, show a disposition to evade payment as much as possible. This fact, as stated by Mr. Wallace, is the best argument in favour of payment by patients, not only in infectious, but in all hospitals, when they are in a position to defray the whole or a portion of the cost of their maintenance and treatment. The circumstances of the time, and the best interests of the population, demand a modification of the present system of free admission in favour of a graduated scale of payments by patients at all hospitals. After all, it is a privilege, and not a hardship for every self-respecting man and woman to pay their way, on all occasions, and to the fullest extent of their power. If the Continental and American system of hospital admission was in force in this country, we should soon hear nothing of hospital abuse, for then the great mass of the people who flock to the hospitals would be much more contented and happier, in many ways, than they are at present.

